# Impact of COVID-19 pandemic on food availability and affordability: an interrupted time series analysis in Ghana

**DOI:** 10.1186/s12889-024-18745-x

**Published:** 2024-05-08

**Authors:** Yoshito Kawakatsu, Ohene Damptey, John Sitor, Ruth Situma, Jevaise Aballo, Mrunal Shetye, Hirotsugu Aiga

**Affiliations:** 1 UNICEF, 4 – 8th Rangoon Close Cantonments, Accra, Ghana; 2 Ministry of Food and Agriculture, Accra, Ghana; 3 United Nations World Food Programme, Accra, Ghana; 4 School of Tropical Medicine and Global Health, Nagasaki University, Nagasaki, Japan

**Keywords:** Food security, COVID-19, Impact, Food price, Food availability, Ghana

## Abstract

**Background:**

In Africa, approx. 675 million people were at risk of food insecurity. COVID-19 pandemic is likely to have exacerbated this situation, by damaging populations’ access to and affordability of foods. This study is aimed at estimating the impacts of the COVID-19 pandemic on availability and prices of essential food commodities at 20 large markets in Ghana.

**Methods:**

Data on food availability and food retail prices collected through weekly market-level data during the period from July 2017 to September 2020 were used in this study. We performed interrupted time-series analyses and estimated the percentage increases between the observed and predicted food prices by food group and by region to assess the impact of COVID-19 pandemic on food prices.

**Results:**

As a result, the impact of COVID-19 on food availability was limited. However, the results of interrupted time-series analyses indicate a significant increase in overall mean food prices in Greater Accra, Eastern and Upper East regions. It was also found that mean price of starchy roots, tubers and plantains significantly increased across regions.

**Discussion:**

The impact of COVID-19 pandemic on food availability and prices was significant but varied by food type and regions in Ghana. Continuous monitoring and responses are critical to maintain food availability and affordability.

**Supplementary Information:**

The online version contains supplementary material available at 10.1186/s12889-024-18745-x.

## Introduction

Globally, more than 700 million people had been infected by coronavirus disease 2019 (COVID-19), with about 1.9 million people having died as of 11th January 2021 [1]. The first COVID-19 case in Sub-Saharan Africa was confirmed in Nigeria on 25th February 2020. The COVID-19 pandemic has already caused considerable hardships in Low and Middle-Income Countries (LMICs), affecting all aspects of populations’ daily life such as health, employment, education, and food security [2–6]. For example, LMICs have reported greater disruptions in essential health services than high-income countries [5]. Employments and income sources of 150 million Africans are at risk during the COVID-19 pandemic [6, 7].

Prior to the onset of the COVID-19 pandemic, approximately two billion individuals globally suffered from moderate or severe food insecurity in 2019 [8]. Among them, 675 million were in Africa [8]. From 2016 to 2018, African countries imported about 85% of their food from countries outside the continent. The COVID-19 pandemic and its prevention measures, such as lockdown restrictions, stay-at-home orders, mass quarantine, and transportation halts, could potentially exacerbate food insecurity situations [9, 10]. According to the preliminary assessment, an additional 83 to 132 million people were estimated to be malnourished by the COVID-19 pandemic, globally [8]. Countries such as Bangladesh, Brazil, China, India, and South Africa reported increased risks of food insecurity due to the COVID-19 pandemic [9–14]. In Bangladesh, the number of households having food insecurity situation increased by 51.7% during the lockdown [14]. In Kenya and Uganda, the proportions of respondents experiencing food insecurity increased by 38% and 44%, respectively, compared to a normal period. Households with low income faced more severe situation of food insecurity in both countries [15]. Two studies in Brazil and China showed variations of the COVID-19 impact on food prices on the market by food commodity and by province [12, 16]. The outbreak may lead to price increases in certain food items as a result of social panic, while the prices of other foods may decrease due to reduced demand during quarantine and limited social activities.

There is a critical need to assess the realities in the field regarding food availability and affordability in Ghana. Therefore, this study aims to estimate the impacts of the COVID-19 pandemic on availability and affordability of essential food commodities in large markets in Ghana, by food group and by region. The hypothesis of this study is that regions with a high number of COVID-19 cases have reduced availability and affordability of essential food commodities in Ghana.

## COVID-19 in Ghana and response

Ghana recorded its first case on 12th March 2020. Since then, the number of confirmed COVID-19 cases increased to about 171,000 as of July 2023 according to WHO Health Emergency Dashboard [17]. The Government of Ghana introduced several measures to prevent COVID-19 transmission from further spreading, ranging from closure of national borders to partial lockdown restriction of some parts of the country, and banning of social and public gatherings [18]. Prior to the closure of national borders, all arriving travelers were compulsorily quarantined for 14 days and tested for a possible COVID-19 infection. A three-week partial lockdown was imposed in Greater Accra Region and Ashanti Region, starting from 30th March 2020. Market traders involved in the production, distribution, and marketing of food were exempted from this partial lockdown, as marketplaces provide essential feeding services to citizens daily. They were also an important income source for sustaining the livelihoods of traders, their families as well as porters and truck pushers. While marketplaces remained open for business, the Ghana Police Service and Ghana Armed Forces were deployed to enforce the lockdown. The Ghanaian authorities requested individuals to purchase food items in their neighborhood markets during the lockdown to avoid heavy traffic and congestion in larger markets [18]. However, the urban poor were often unable to comply with the lockdown directive. Additionally,, staying home for three weeks was difficult for most of the poor population, leading to no access to affordable food or completely running out of money to buy food. In response, the Ghanian government and private organizations distributed food packages to households and communities. Moreover, the state covered water bills for all Ghanaians for April to June 2020 [18]. Also, during this period, teams dispatched by the Ghana Health Service (GHS) conducted extensive testing and contact tracing within the confinement areas. For various reasons (e.g., hardships on people’s life due to suspension of daily activities and resultant lack of compliance), the lockdown restriction was relaxed on 24th April. While announcing the easing of the restriction, the President warned that there would be an expanded programme of testing and strict enforcement of social distancing and hygiene protocols including compulsory wearing of face masks. Numerous studies have reported on the comprehensive and detailed COVID-19 responses in Ghana [19–22].

## Methods

This study conducted an interrupted time series analysis, utilizing the weekly market surveillance, to estimate the impacts of the COVID-19 pandemic on availability and affordability of essential food commodities in large markets in Ghana.

### Data collection

The primary data source for this study was the dataset on food availability and retail prices of essential food commodities from weekly market surveillance, conducted during the period from 22nd July 2017 to 30th September 2020 by the Ministry of Food and Agriculture (MoFA). A total of 21 essential food commodities were traced in the surveillance system: millet, soya bean, white maize, yellow maize, cowpea, sorghum, rice, cassava, white yam, puna yam, cocoyam, apentu plantain (local name), apem plantain(local name), gari (i.e., a fine to coarse granular flour made from cassava tubers), onion, tomato, chicken, eggs and their products, fish (mackerel), dried pepper, and fresh pepper. These commodities are further categorized into eight food groups as defined by the Food and Agriculture Organization of the United Nations [23]. Table 1 shows the essential food commodities by food group. Food retail prices were measured in Ghanaian Cedi (GH₵: the local currency) per kg.

**Table 1:** Essential food commodities by food group

Food groups	No. of food items	Name of commodities monitored
Legumes and their products	5	Millet, Soya bean, Maize (White and Yellow), Cowpea
Cereals and their products	2	Sorghum, Rice
Starchy roots, tubers, plantains and their products	7	Cassava, Yam (White and Puna), Cocoyam, Gari, Plantain (Apentu and Apem)
Vegetables and their products	2	Onion, tomato
Meat, poultry and their products	1	Chicken
Eggs and their products	1	Egg
Fish and their products	1	Fresh fish (Mackerel)
Miscellaneous	2	Dried and Fresh pepper

The food monitors and data collection officers involved in this surveillance are from MoFA and the Ministry of Local Government and Rural Development. They were recruited based on their experience in data collection, fluency in the local language and familiarity with the local terrain.

**Fig. 1:**
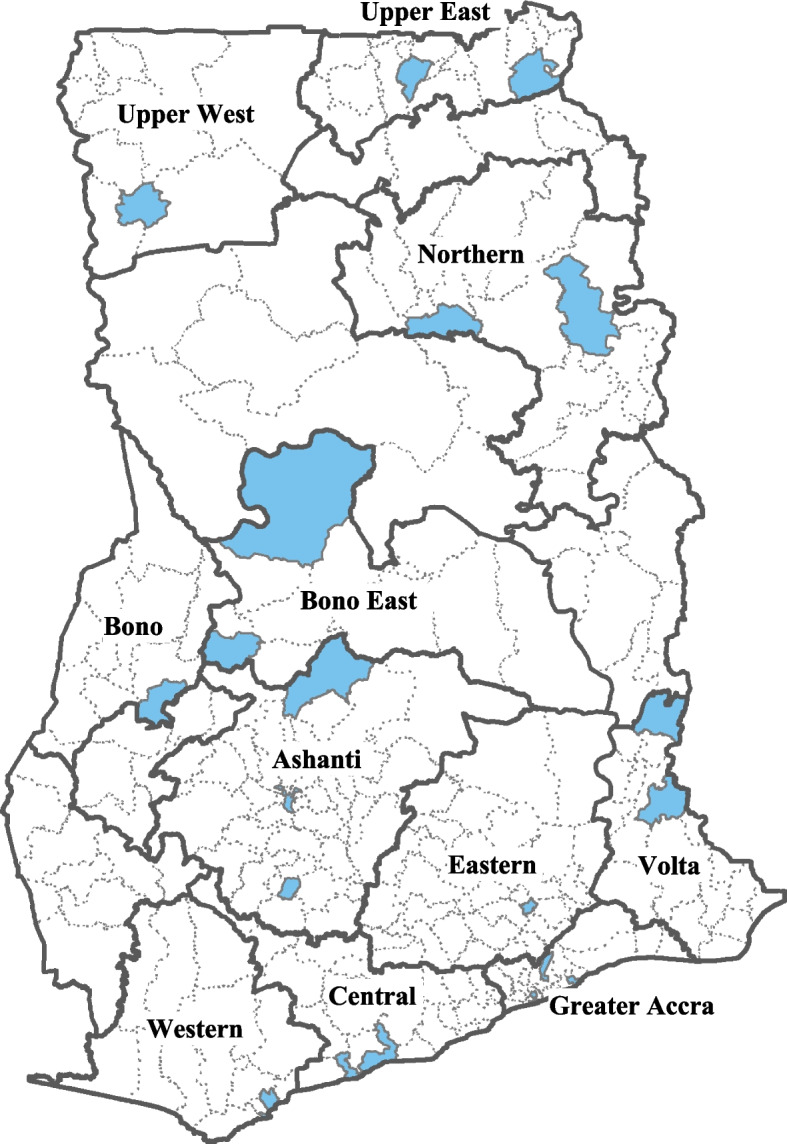
Districts in which 20 large markets are located

They are trained in the use of digital tools and in administering questionnaires. They have weekly collected data on the availability and mean prices of the 21 essential food commodities at 20 large markets out of approximately 170 district and regional markets throughout Ghana, as part of its weekly market surveillance system. These 20 markets are located in 11 of 16 regions of Ghana. These 11 regions include Ashanti, Bono, Bono East, Central, Eastern, Greater Accra, Northern, Upper East, Upper West, Volta and Western Regions. Figure 1 shows the locations of 20 markets in the districts of Ghana. This food monitoring was conducted as long as food monitors were readily available and the target markets were accessible, even during the COVID-19 pandemic. At the markets, at least 3 stores/market were randomly selected using simple random selection methodology. They checked the prices and quantities in the stores/markets and the timeliness and frequency of replenishment of the stock when it runs out, using the digital tools. The mean price of each essential food commodity at each market was calculated through simple average calculations.

We analyzed the weekly food availability and food retail price data collected between 22nd July 2017 and 30th September 2020 (i.e., 164 weeks in total). Note that the number and types of essential food commodities were nationally standardized and consistent across the 20 markets during the period. Data collection was not possible during the 1st and 2nd week of April due to the lockdown. In addition, there was some missing data accounting for 8.2% of the total required number of observations, due to the temporary inability to employ an adequate number of capable food monitors. The percentage of missing data before the COVID-19 pandemic was 9.6%, while during the pandemic, it was 5.5%. The mixed-models using maximum likelihood estimation accounted for missing data [24].

### Data analysis

The outcomes in this study were availability and retail prices of the essential food commodities. In this study, food availability is defined as a dichotomous variable (i.e., whether a given food commodity is available or not in markets). Food retail price is defined as a continuous variable in form of amount of GH₵ without considering inflation and deflation adjustments. This is because the exchange rate of GH₵ into United States Dollars (USD) was stable during the study period from March to December 2020 (i.e., GH₵ 1 equivalent to USD 0.17). Moreover, COVID-19 impacts were divided into two phases: (i) during the initial spread of COVID-19 (12th March ─ 31st July 2020); and (ii) after the peak of first wave of COVID-19 (1st August ─ 30th September 2020). This is because the number of newly confirmed COVID-19 cases started declining on 1st August 2020 in Ghana [17]. Thus, two continuous variables related to COVID-19 pandemic were created. One is *COVID1* defined as the number of weeks having passed since the first confirmed COVID-19 case was detected on 12th March 2020. The other is *COVID2* defined as the number of weeks having passed since the peak of first wave of COVID-19 on 1st August 2020.

For food availability, a descriptive analysis was performed to show the change in the proportion of available ones to the total number of essential food commodities (= 21) between before and during the COVID-19 pandemic. For food retail prices, in addition to descriptive analyses, two types of interrupted time-series analyses (ITS) (i.e., food-group-based model and region-based model) were performed, using the mixed-effects linear models. ITS is the strongest quasi-experimental method in causal inference since it can control long-term time trends, account for observation-level bias and clustering and conduct stratified analyses of subpopulations. We accounted for autocorrelation by including autoregressive errors in both models. A random effect was added in each model to account for data clustering of markets. In addition, the models’ time variable was designed to capture and isolate the impacts attributable to the COVID-19 pandemic from those associated with general inflationary trends. The food-group-based model includes the interaction terms between the variables of COVID-19 impacts (i.e., *COVID1* and *COVID2*) and food group, while region-based model includes the interaction terms between the variables of COVID-19 impact and regions. Systematic components of the two models are shown below.

Food-group-based model:

1 $$\begin{array}{c}{Food \,Price}_{ijt}={(\beta }_{0}+{\theta }_{ij})+{\beta }_{1} Tim{e}_{t}+{\beta }_{2-11}Month+{\beta }_{12-18}Food\, group_{i}{+\beta }_{19-28}{Region}_{j}+{\beta }_{29}{COVID1}_{ijt}+{\beta }_{30}{COVID2}_{ijt}+{\beta }_{31-37}Food \,group_{i}\times Tim{e}_{t}+{\beta }_{38-44}Food\, group_{i}\times {COVID1}_{ijt}+{\beta }_{43-49}Food \,group_{i}\times {COVID2}_{ijt}\\ {\theta }_{ij}\sim N\left(0,{\sigma }_{0}^{2}\right)\end{array}$$

Region-based model:

2 $$\begin{array}{c}{Food\, Price}_{ijt}={(\beta }_{0}+{\theta }_{ij})+{\beta }_{1} Tim{e}_{t}+{\beta }_{2-11}Month+{\beta }_{12-18}Food \,group_{i}{+\beta }_{19-28}{Region}_{j}+{\beta }_{29}{COVID1}_{ijt}+{\beta }_{30}{COVID2}_{ijt}+{\beta }_{31-40}{Region}_{j}\times Tim{e}_{t}+{\beta }_{41-50}{Region}_{j}\times {COVID1}_{ijt}+{\beta }_{51-60}{Region}_{j}\times {COVID2}_{ijt}\\ {\theta }_{ij}\sim N\left(0,{\sigma }_{0}^{2}\right)\end{array}$$

where,

**Table:** 

*Food Price*_*itc*_	Food retail price (GH₵) of i^th^ food commodity per kg in j^th^ market at t^th^ week
*β*_*0*_ + *θ*_*0ij*_	Intercept of i^th^ food commodity in j^th^ market at time zero
*Time*	Number of weeks since 22nd July 2017 (min 0 ─ max 164) in the reference region
*Month*	Dummy variables for 11 months of calendar year (Reference category: January)
*Food group*	Dummy variables of seven groups (Reference category: Legumes and their products)
*Region*	Dummy variables of ten regions (Reference category: Bono East region)
*COVID1*	Number of weeks having passed since the first confirmed COVID-19 case was detected on 12th March 2020, which captures the progression of the COVID-19 pandemic from its onset
*COVID2*	Number of weeks having passed since the peak of the first wave of COVID-19 on 1st August 2020, which enables to distinguish the pandemic’s continuing effects on food prices after the peak of the first wave
*Food group* × *Time*	Interaction terms between *Food group* and* Time*
*Food group* × *COVID1*	Interaction terms between *Food group* and* COVID1*
*Food group* × *COVID2*	Interaction terms between *Food group* and* COVID2*
*Region* × *Time*	Interaction terms between *Region* and* Time*
*Region* × *COVID1*	Interaction terms between *Region* and* COVID1*
*Region* × *COVID2*	Interaction terms between *Region* and* COVID2*

By applying the two models, we predicted prices of the essential food commodities with and without COVID-19 pandemic from 12th March to 30th September 2020. We hypothesized that the COVID-19 pandemic did not occur as a counterfactual. It means that the fixed effects and interaction terms of *COVID1 and COVID2* are set as 0. We further calculated the percentage increases between the observed and predicted food prices by food group and by region. Supplement files 1 and 2 show the model fitting of the two models. All the statistical analyses were performed by using *R* (Foundation for Statistical Computing, Vienna, Austria).

## Results

### Food availability

**Fig. 2:**
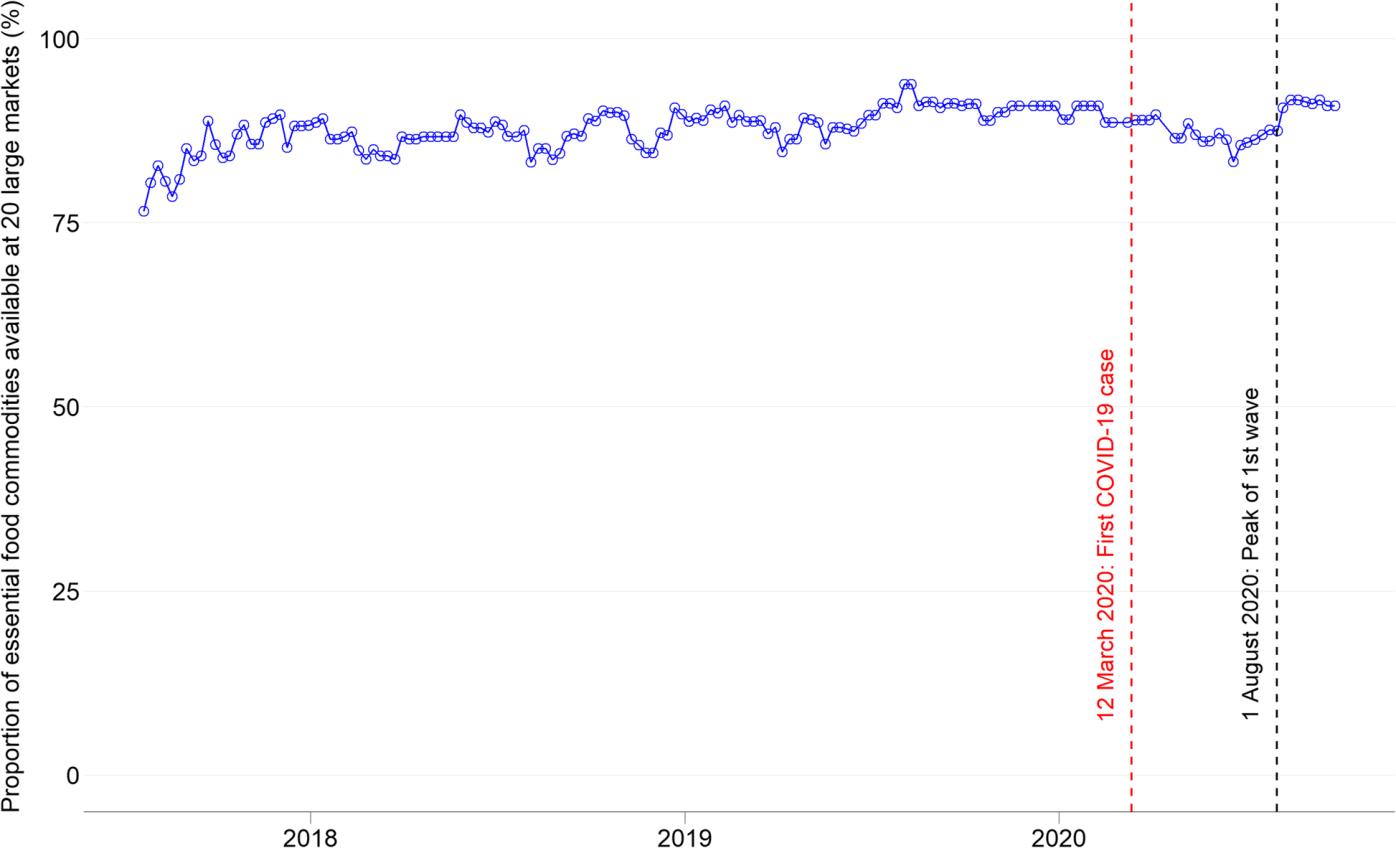
Changes in the proportion of available essential food commodities

Figure 2 shows time series changes in availability of the essential food commodities at 20 study markets in 11 regions from 22nd July 2017 to 30th September 2020. The proportions of available one to total number of the essential food commodities (= 21) before and after March 2020 were 87.9% and 88.1% on average, respectively. Overall, 88% of 21 essential food commodities (i.e., 18–19 commodities) continued to be available at the 20 market regardless of occurrence of the COVID-19 pandemic. Also, Supplement file 3 provides the change of proportion of food availability by food group.

### Food affordability by food group

**Fig. 3:**
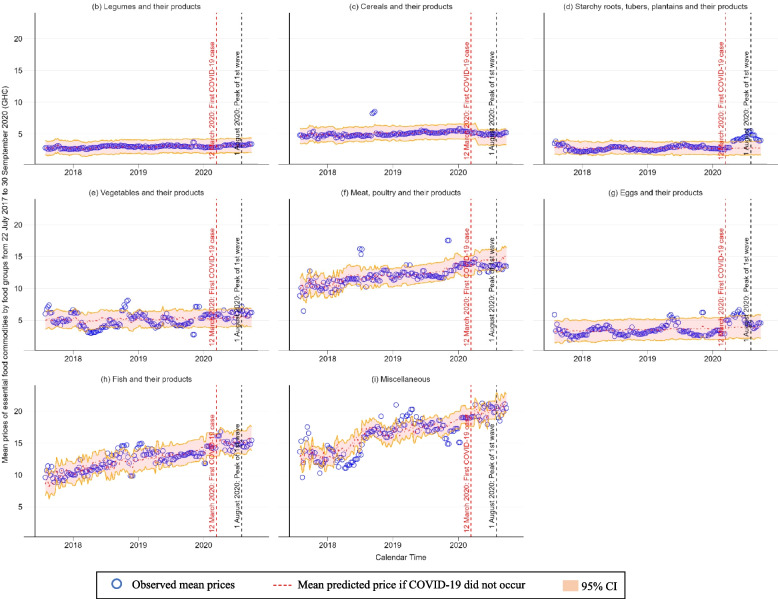
Changes in mean observed and predicted prices of essential food commodities by food group

Figure 3 shows the changes in weekly mean retail prices of overall and individual food groups from July 22nd^, 2017^, to 30th September 2020. Overall mean retail price of all the eight food groups continued to increase after detection of the first confirmed COVID-19 case on 12th March 2020 the peak of first wave of COVID-19 on 1st August (Fig. 3 (a)). After the peak, the mean price of overall food groups decreased (Fig. 3 (a)). This overall food price reduction was primarily attributed to greater reduction in mean prices of starchy food group (starchy roots, tubers, plantains and their products) and eggs and their products (Fig. 3 (d) and (g)). The overall mean price as of 30th September was higher systematically than that as of 12th March 2020, the date of detection of the first confirmed COVID-19 case (Fig. 3 (a)). Food prices at the retail level also showed variations across different food groups, as shown in Fig. 3 (b)-(i).

Table 2 presents the result of the interrupted-time series analysis with the interaction terms between COVID-19 phases and food groups. Being accounted for regions, seasonality, and inflation over time, a significant increase in the mean prices of the starchy food group during the initial five-month spread of COVID-19 was observed. Subsequently, during the two-month period following the peak of the first wave of COVID-19, there was a significant decrease in the prices of this food group. Table 3 presents the percentage increases by food group between observed and predicted prices under the counterfactual assumption that the COVID-19 pandemic had not occurred. During the initial spread of COVID-19 pandemic from 12th March to 31st July 2020, observed mean food price of starchy food group was 50.5% higher (95% CI: 8.9 – 143.4) than predicted one. Despite its drop during the two-month period after the peak of first wave of COVID-19, mean price of starchy food group was 59.4% higher (95% CI: 15.4 – 157.6) than predicted one. Mixed results of percentage increases were found in the other food groups, too. Also, Supplement file 4 provides full results of interrupted time-series analysis with the interaction between food group and COVID-19 impacts.

**Table 2:** Results of interrupted time-series analysis with the interaction between food groups and COVID-19 impacts

	**Coefficients**^**a**^	**SE**
**Reference group: Legumes and their products**		
COVID1	0.004	0.018
COVID2	0.009	0.054
**Interaction between COVID-19 impacts and food groups**		
Cereal × COVID1	0.029	0.030
Cereal × COVID2	-0.033	0.088
Starchy × COVID1	0.123^b^	0.023
Starchy × COVID2	-0.284^b^	0.070
Vegetable × COVID1	0.041	0.033
Vegetable × COVID2	-0.075	0.100
Meat × COVID1	-0.068	0.043
Meat × COVID2	0.037	0.130
Egg × COVID1	0.051	0.043
Egg × COVID2	-0.123	0.13
Fish × COVID1	-0.026	0.044
Fish × COVID2	0.019	0.134
Miscellaneous × COVID1	-0.020	0.034
Miscellaneous × COVID2	-0.067	0.103

^a^Controlled by time, seasonality, and Regions

^b^ < .001

**Table 3:** Percentage increases in food prices by food group between observed price and predicted price under the counterfactual assumption that COVID-19 had not occurred

**Food group**	**Initial-spread of COVID-19** (16th March to 31st July 2020)			**After the first wave peak** (1st August to 30th September 2020)		
	**% increase**	**95% CI**		**% increase**	**95% CI**	
		**Lower**	**Upper**		**Lower**	**Upper**
Legumes and their products	1.48	-25.03	56.99	4.52	-22.71	61.34
Cereals and their products	7.98	-14.39	46.17	14.83	-9.73	57.73
Starchy roots, tubers, plantains and their products	50.48	8.90	143.39	59.39	15.39	157.64
Vegetables and their products	9.79	-12.58	47.56	13.78	-9.56	53.33
Meat, poultry and their products	-5.09	-14.54	6.72	-8.98	-18.24	2.65
Eggs and their products	15.58	-20.08	108.74	18.74	-18.19	116.43
Fish and their products	-1.65	-12.16	11.71	-2.53	-12.98	10.77
Miscellaneous	-0.88	-7.50	6.76	-3.16	-9.40	4.00

### Food affordability by region

**Fig. 4:**
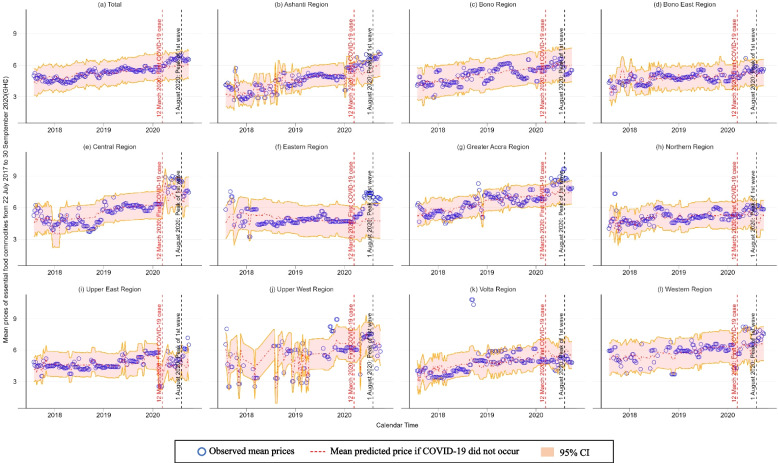
Changes in mean observed and predicted prices of essential food commodities by region

Figure 4 shows the changes in weekly mean overall food prices of each region from July 22nd^, 2017^, to 30th September 2020. In Greater Accra Region where the national capital is located, food prices significantly increased during the initial spread of COVID-19 and decreased during the two-month period after the peak of first wave of COVID-19. COVID-19 pandemic is likely to have significantly increased the food prices also in Eastern Region during its five-month initial spread. Interestingly, food prices in the Upper East Region significantly increased only during the two-month period after the peak of first wave of COVID-19.

**Fig. 5:**
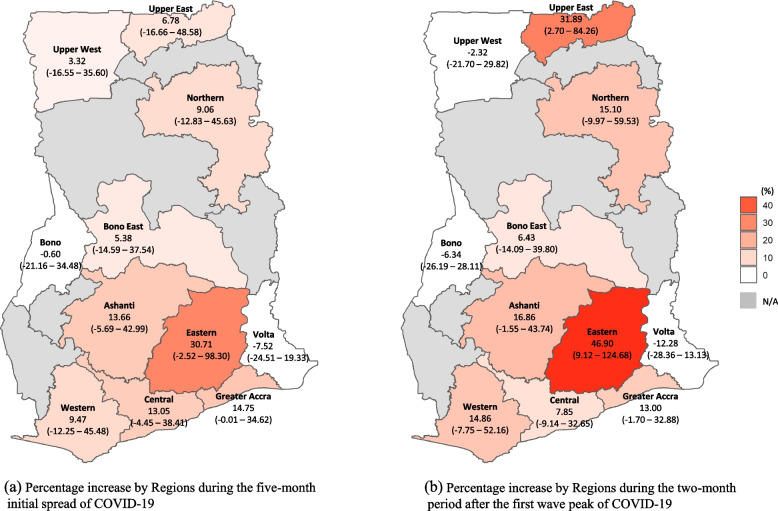
Percentage increases in food prices by region between observed price and predicted price under the counterfactual assumption that COVID-19 had not occurred

Figure 5 presents the percentage increases by region between observed and predicted prices under the counterfactual assumption that the COVID-19 pandemic had not occurred. The overall mean price of all the food groups in Eastern Region during the five-month initial spread of COVID-19 was 30.7% (95% CI: -2.5 – 98.3) higher than the predicted one. Similarly, overall mean food price during the two-month period after the peak of first wave of COVID-19 was 31.9% (95% CI: 2.7 – 84.3) and 13.0% (95% CI: -1.7 – 32.9) higher than predicted ones in Upper East Region and Greater Accra Region, respectively. Also, Supplement file 5 provides full results of interrupted time-series analysis with the interaction between regions and COVID-19 impacts.

## Discussion

### Food availability

Though the greater proportion of essential food commodities available on the market (approx. 88%) was sustainably ensured regardless of the COVID-19 pandemic, there was a slight drop at the beginning of the initial spread of COVID-19 (i.e., from mid-March to mid-June 2020). Note that this fluctuation between mid-March and mid-June 2020 aligns with the seasonal trend in the previous two years before the COVID-19 pandemic (Fig. 2). An increase in the proportion of essential food commodities after mid-June 2020 also aligned with the seasonal trend during the last two years. Thus, these changes in food availability are more likely attributed not to the COVID-19 pandemic but to normal agricultural seasonal activities in Ghana (i.e., sowing and growing in March – June; and harvesting in July—September).

The results of our study indicate that the impact of the COVID-19 pandemic on food availability was limited in Ghana, likely due to the well-established system of local production of essential food commodities. Generally, food production in Ghana increased in 2019 compared to 2018 [25]. Although Ghana largely depends on imports for its rice supply (60%) [26], rice is storable for more than one year. Moreover, it appears that the COVID-19 pandemic did not trigger major behavioral changes among farmers in their agricultural activities in 2020 (e.g., sowing, growing, and harvesting). Although the lockdown restriction affected supplies of agricultural inputs across the country for a short period, the production process of major staple foods was not significantly affected. Farmers continued to plant during the major sowing season for major crops like maize and millet (June to August) after the lockdown restriction was relaxed. Ghana’s Strategic Policy of Investing in Food and Jobs ensured that farmers continued to receive agricultural inputs such as certified seeds, fertilizers, mechanization, and extension services. In addition, the government did not apply the border closure to goods, supplies, and cargo, thus ensuring a continuous flow of agricultural commodities [18].

### Food affordability by food group

This study found a significant increase in the food retail prices of starchy food groups (such as starchy roots, tubers, plantains, and their products), particularly during the initial five-month spread of COVID-19. Despite the significant price drop during the two months after the peak of first wave of COVID-19, the mean prices remained higher than those predicted under the counterfactual assumption that the COVID-19 pandemic had not occurred.

Starchy roots, tubers, and plantains, which are consumed at a high rate in Ghana, are locally produced but have a high rate of perishing, while cereals and legumes can be stored for a longer period. The movement restrictions and the economic slowdown caused by the lockdown made it challenging for farmers to regularly access their farms. Consequently, a reduced total food supply and a higher rate of perishability likely contributed to higher prices of starchy roots, tubers, and plantains. In addition, the populations’ panic buying behaviors and changes in food consumption patterns led to an unexpected increase in their demands for main staple foods [2, 27]. After the first wave of the COVID-19 pandemic in Ghana, approximately a total of 42,000 and 12,000 employees were laid off in May–June and August–September 2020, respectively (Ghana Statistical Service, 2020). India and Pakistan also reported a reduction of household income around March and April [2]. Although income and demand for foods are closely linked in developing countries, prices of staple foods remained high. Households tended to purchase relatively cheaper staple foods than more expensive but more nutrient-dense foods. Food consumption pattern during the COVID-19 pandemic under the difficult economic situation should be assessed to prevent micronutrient deficiencies among the poor population.

The increase in prices of starchy food groups could partially be attributed to their continued usual export to neighboring countries such as Niger, Mali, and Burkina Faso. Cassava, in particular, has been rapidly gaining significant commercial value in Ghana due to its increased demand from the Chinese-operated starch factories springing up in major industrial parks across the country. Thus, the COVID-19 pandemic, among several other possible factors, is likely to have contributed to the rising prices of these starchy food groups. Supplement file 4 provides the full results of interrupted time-series analysis with the interaction between food groups and COVID-19 impacts.

### Food affordability by region

Market trading in Ghana heavily relies on imported goods and products from neighboring countries such as Burkina Faso and Nigeria as well as other countries such as China and India. Amidst the supply shortage caused by the global surge of COVID-19 cases, sharp increases in food prices were observed in urban markets across the country [18].

In this study, we observed that mean food prices in Greater Accra Region significantly increased during the initial spread of COVID-19, but subsequently fell in the two months following the peak of the first wave. The increase in the prices of starchy food groups largely contributed to the changes in overall mean food prices (see Supplement file 6). Greater Accra Region was Ghana’s greatest epicenter of the COVID-19 pandemic, followed by Ashanti Region. Both regions, which underwent partial lockdowns, experienced similar rapid increases in food prices, particularly in starchy food groups (Fig. 4 (b), (g) and Supplement file 6). However, the results of our study indicate that the overall mean food price in Ashanti Region was not significantly affected (Table 4). During the initial spread of COVID-19, some consumers likely increased demand for foods than usual for hoarding purposes. This is a typical means of contingency planning and emergency preparedness for reducing the risks of infection by refraining from going shopping [28] and for hoarding foods [29]. Such panic buying and hoarding of non-perishable foods during the initial spread of COVID-19 were also reported in the United States and United Kingdom [30, 31]. While evidence of panic buying during the COVID-19 pandemic in Sub-Saharan Africa was limited, a sharp increase in food prices due to panic buying, disruptions in internal and external trades, and changes in marketing activities during the outbreak of Ebola virus disease were reported in Guinea, Liberia, and Sierra Leone [32].

**Table 4:** Results of interrupted time-series analysis on food prices with the interaction between regions and COVID-19 impacts

	**Coefficients**^**a**^	**SE**
**Reference group: Bono East region**		
COVID1	0.026	0.026
COVID2	-0.058	0.080
**Interaction between COVID-19 impacts and Regions**		
Ashanti × COVID1	0.041	0.034
Ashanti × COVID2	-0.065	0.108
Bono × COVID1	-0.029	0.044
Bono × COVID2	-0.002	0.136
Central × COVID1	0.052	0.041
Central × COVID2	-0.193	0.115
Eastern × COVID1	0.108^b^	0.045
Eastern × COVID2	-0.142	0.140
Greater Accra × COVID1	0.075^b^	0.034
Greater Accra × COVID2	-0.235^b^	0.103
Northern × COVID1	0.016	0.042
Northern × COVID2	0.006	0.120
Upper East × COVID1	-0.002	0.038
Upper East × COVID2	0.231^b^	0.114
Upper West × COVID1	-0.004	0.046
Upper West × COVID2	-0.083	0.144
Volta × COVID1	-0.063	0.038
Volta × COVID2	0.094	0.114
Western × COVID1	0.030	0.044
Western × COVID2	-0.017	0.137

^a^Controlled by time, seasonality, and food groups

^b^ < .05

Market traders in the lockdown areas (i.e., Greater Accra and Ashanti regions) were exempted from movement restriction. However, some security officers restricted the movement of traders who were permitted to bring the items from the central markets to neighborhood markets [18]. While shoppers reported that the traders had deliberately increased food prices to take advantage of panic buying, traders argued that suppliers had increased the prices [18]. Further investigation is required to clarify the mechanism of food price increases during disease pandemics and emergencies. A previous report noted ed similar increases in food prices and its reasons in Guinea, Liberia, and Sierra Leone during the outbreak of Ebola virus disease [32]. The situation during the COVID-19 pandemic would be more complex compared to the Ebola outbreak, as the pandemic has resulted in global trading restrictions and shortages in supplies.

The Eastern Region, which borders both the Greater Accra Region and Ashanti Region that were two most suffered regions by the COVID-19 related restrictions, experienced a unique situation. The abrupt increase in food price in these neighboring regions, triggered by the restrictions, likely incentivized farmers and food suppliers in Eastern Region to sell more foods in Greater Accra Region and Ashanti Region to maximize their sales and profits. Consequently, this would lead to food shortages and rising food prices within the Eastern Region itself. Moreover, people in the lockdown regions were required to purchase food items from their neighborhood markets during the lockdown. It would result in increasing the demand for food items and their prices in the Eastern region [18]. Some people in Greater Accra Region and Ashanti Region, in which the COVID-19-related restrictions were enforced, were likely to migrate to other regions, including the Eastern Region, due to fears of infection. Fear of infection has been identified as a significant driver of population movement during the pandemic [33].

During the five-month initial spread of COVID-19, the number of confirmed cases in the northern part of Ghana was limited. Therefore, the Upper East Region, a major food-producing area in the northern part, did not implement lockdowns or other forms of restrictions. This allowed local food prices in the region to remain stable during this period. However, once restrictions were relaxed in Greater Accra Region and Ashanti Region, food suppliers likely began transporting food to these two most affected regions in search of greater sales and profits. Thus, during the two-month period after the peak of first wave of COVID-19, the Upper East Region would presumably experience intra-regional food shortages and, thereby, rapid food price increases due to increased demands from Greater Accra Region and Ashanti Region.

In addition to increases of food retail prices in some regions of Ghana, the COVID-19 pandemic negatively impacted economic growth, employment, and income [7, 10]. In Ghana, 46% and 28% of business firms reported reducing wages in May/June and August/September 2020, respectively [34]. Also, approximately a total of 42,000 and 12,000 employees were laid off in May–June and August–September 2020, respectively [34]. Consequently, it is evident that the populations’ food-purchasing power was largely reduced. Therefore, the combination of increases in food prices, income loss, and wage reductions synchronously impaired and further exacerbated populations’ ability to afford food. High food prices, especially for the most vulnerable population, can lead to reduced food consumption and poor food security indicators [35]. A rapid assessment in Kenya and Uganda reported that households with low income were more likely to experience food insecurity during the COVID-19 pandemic [15].

## Limitations

There are some limitations in this study. First, this study analyzed the data of food availability and food retail prices collected exclusively at 20 large markets. Therefore, there is a certain limitation in the national representation of the study results. Food availability and food retail prices at smaller markets might have been affected by the COVID-19 pandemic. However, it is important to note that changes in food availability and food prices at large markets are likely to have influenced those at smaller markets. Further investigation is recommended, targeting smaller markets, primarily in rural areas. Second, 20 large markets were not randomly but purposively selected. While admitting some possible sampling bias, purposively sampling 20 central markets in major cities or districts should help the study ensure a certain level of representativeness. Third, there was missing data in our dataset. We assumed that data missing randomly occurred in our analysis. Also, while the overall availability of essential food commodities remained high over time, unavailability of food, even if infrequent, might influence the results of food price analyses due to supply–demand imbalances. Factors such as market speculation, consumer behavior changes, and localized supply chain disruptions could contribute to food availability and price variations. These aspects underscore the complex interplay between food availability and pricing mechanisms. Since individual data were not collected from households and farmers in this study, there are limitations in interpreting data in the context of food affordability. Future studies are necessary to assess more detailed household food security (i.e., households’ or individuals’ access to, affordability, and consumption of foods) for more precisely estimating the impact of the COVID-19 pandemic.

## Conclusion

While the impact of the COVID-19 pandemic on food availability was limited in Ghana, our study revealed a significant increase in the mean price of starchy food group due to the COVID-19 pandemic. Furthermore, mean food prices of all the essential food commodities in the Greater Accra Region, Eastern Region, and Upper East Region significantly increased. Given these findings, it is recommended that further investigations and continued monitoring be conducted to more precisely understand how households and farmers are coping with the prolonged impact of COVID-19 pandemic. Such research should aim to provide a more comprehensive understanding of the dynamics of food availability and affordability. Our finding also suggests that there is a need for policies that address the volatility in food prices, particularly in regions that have experienced significant increases. Strategies could include supporting local food, implementing price control measures for essential food items, and providing targeted financial assistance to the most affected households. Furthermore, understanding the coping mechanisms of households and farmers will help in developing targeted interventions to assist those most impacted by the pandemic and in preparing for future infectious disease outbreaks.

### Supplementary Information

Supplementary Material 1.

Supplementary Material 2.

Supplementary Material 3.

Supplementary Material 4.

Supplementary Material 5.

Supplementary Material 6.

## Data Availability

The datasets analysed during the current study are not publicly available due to the Ghanian governmental regulation but are available from the corresponding author on reasonable request with agreement from the Ministry of Food and Agriculture, Ghana.

## References

[1] WHO Coronavirus Disease (COVID-19) Dashboard. https://covid19.who.int/.

[2] Workie E, Mackolil J, Nyika J, Ramadas S (2020). Deciphering the impact of COVID-19 pandemic on food security, agriculture, and livelihoods: A review of the evidence from developing countries. Curr Res Environ Sustain.

[3] Marinoni G, van’t Land H, Jensen T (2020). The impact of COVID-19 on higher education around the world. IAU global survey report.

[4] Donohue JM, Miller E (2020). COVID-19 and School Closures. JAMA.

[5] World Health Organization (2020). Pulse survey on continuity of essential health services during the COVID-19 pandemic.

[6] Jayaram K, Leke A, Ooko-Ombaka A, Sun YS. Finding Africa’s path: shaping bold solutions to save lives and livelihoods in the COVID-19 crisis. NYC: McKinsey and Company; 2020.

[7] Calderon C, Kambou G, Djiofack CZ, Korman V, Kubota M, Catalina CC (2020). Africa's pulse, No. 21, Spring 2020 : An analysis of issues shaping Africa’s economic future.

[8] Food and Agriculture Organization of the United Nations, IFAD, UNICEF, WFP, WHO. The State of Food Security and Nutrition in the World (2020). Transforming food systems for affordable healthy diets.

[9] Reardon T, Mishra A, Nuthalapati CSR, Bellemare MF, Zilberman D (2020). Covid-19’s disruption of India’s transformed food supply chains. Econ Pol Wkly.

[10] Arndt C, Davies R, Gabriel S, Harris L, Makrelov K, Robinson S, Levy S, Simbanegavi W, van Seventer D, Anderson L (2020). Covid-19 lockdowns, income distribution, and food security: An analysis for South Africa. Glob Food Sec.

[11] Pu M, Zhong Y (2020). Rising concerns over agricultural production as COVID-19 spreads: Lessons from China. Glob Food Sec.

[12] de Paulo FD, de Araújo FF (2020). Will COVID-19 affect food supply in distribution centers of Brazilian regions affected by the pandemic?. Trends Food Sci Technol.

[13] Mishra K, Rampal J (2020). The COVID-19 pandemic and food insecurity: A viewpoint on India. World Dev.

[14] Hamadani JD, Hasan MI, Baldi AJ, Hossain SJ, Shiraji S, Bhuiyan MSA, Mehrin SF, Fisher J, Tofail F, Tipu SMMU (2020). Immediate impact of stay-at-home orders to control COVID-19 transmission on socioeconomic conditions, food insecurity, mental health, and intimate partner violence in Bangladeshi women and their families: an interrupted time series. Lancet Glob Health.

[15] Kansiime MK, Tambo JA, Mugambi I, Bundi M, Kara A, Owuor C (2021). COVID-19 implications on household income and food security in Kenya and Uganda: Findings from a rapid assessment. World Dev.

[16] Yu X, Liu C, Wang H, Feil J-H (2020). The impact of COVID-19 on food prices in China: evidence of four major food products from Beijing, Shandong and Hubei Provinces. China Agricultural Econ Rev.

[17] WHO Health Emergency Dashboard. https://covid19.who.int/region/afro/country/gh.

[18] Asante LA, Mills RO (2020). Exploring the socio-economic impact of COVID-19 pandemic in marketplaces in urban Ghana. Afr Spectr.

[19] Sibiri H, Prah D, Zankawah SM (2021). Containing the impact of COVID-19: Review of Ghana's response approach. Health Policy Technol.

[20] Nimako BA, Baiden F, Awoonor-Williams JK (2020). Towards effective participation of the private health sector in Ghana's COVID-19 response. Pan Afr Med J.

[21] [Case Study] Ghana’s multifarious response to COVID-19: through a citizen’s lens. https://www.ingsa.org/covidtag/covid-19-commentary/asantewah-nkansah-ghana/.

[22] Dzansi J. Ghana lifts the lockdown: has the government reneged on its commitment to contain COVID-19 at all costs?, vol. 2021. London: International Growth Centre; 2020.

[23] Food and Agriculture Organization of the United Nations, FHI 360 (2016). Minimum dietary diversity for women: a guide for measurement.

[24] Allison P (2012). Handling missing data by maximum likelihood.

[25] Food and Agriculture Organization of the United Nations (2020). GIEWS country brief Ghana.

[26] Archibald D, Taylor J. Ghana grain and feed annual 2019 annual report. Washington, DC: USDA Foreign Agriculture Service; 2019.

[27] Loxton M, Truskett R, Scarf B, Sindone L, Baldry G, Zhao Y (2020). Consumer behaviour during crises: Preliminary research on how coronavirus has manifested consumer panic buying, herd mentality, changing discretionary spending and the role of the media in influencing behaviour. J Risk Financ Manag.

[28] Cranfield JAL (2020). Framing consumer food demand responses in a viral pandemic. Canadian J Agri Economics/Revue canadienne d'agroeconomie.

[29] Beard-Knowland T (2020). The impact of covid-19 on how we eat.

[30] Baker SR, Farrokhnia RA, Meyer S, Pagel M, Yannelis C (2020). How does household spending respond to an epidemic? Consumption during the 2020 COVID-19 Pandemic. Rev Asset Pricing Stud.

[31] Chronopoulos DK, Lukas M, Wilson JOS. Consumer Spending Responses to the COVID-19 Pandemic: An Assessment of Great Britain. 2020. Available at SSRN: https://ssrn.com/abstract=3586723.

[32] Food and Agriculture Organization of the United Nations (2014). Grave food security concerns following the Ebola outbreak in Liberia, Sierra Leone and Guinea. global information and early warning system on food and agriculture (GIEWS).

[33] Campbell L (2017). Learning from the ebola response in cities: Population movement. ALNAP Working Paper.

[34] Ghana Statistical Service (2020). How COVID-19 is affecting firms in Ghana: Results from the Business Tracker Survey – Wave 2.

[35] Gustafson DJ (2013). Rising food costs & global food security: key issues & relevance for India. Indian J Med Res.

