# Supplementary material for: Impact of COVID-19 pandemic on food availability and affordability: an interrupted time series analysis in Ghana
Source: BMC Public Health. 2024 May 8;24:1268. doi: 10.1186/s12889-024-18745-x (PMC11080309; doi:10.1186/s12889-024-18745-x)
Supplement: Supplementary file 1 — Supplementary Material 1. [file 12889_2024_18745_MOESM1_ESM.docx]

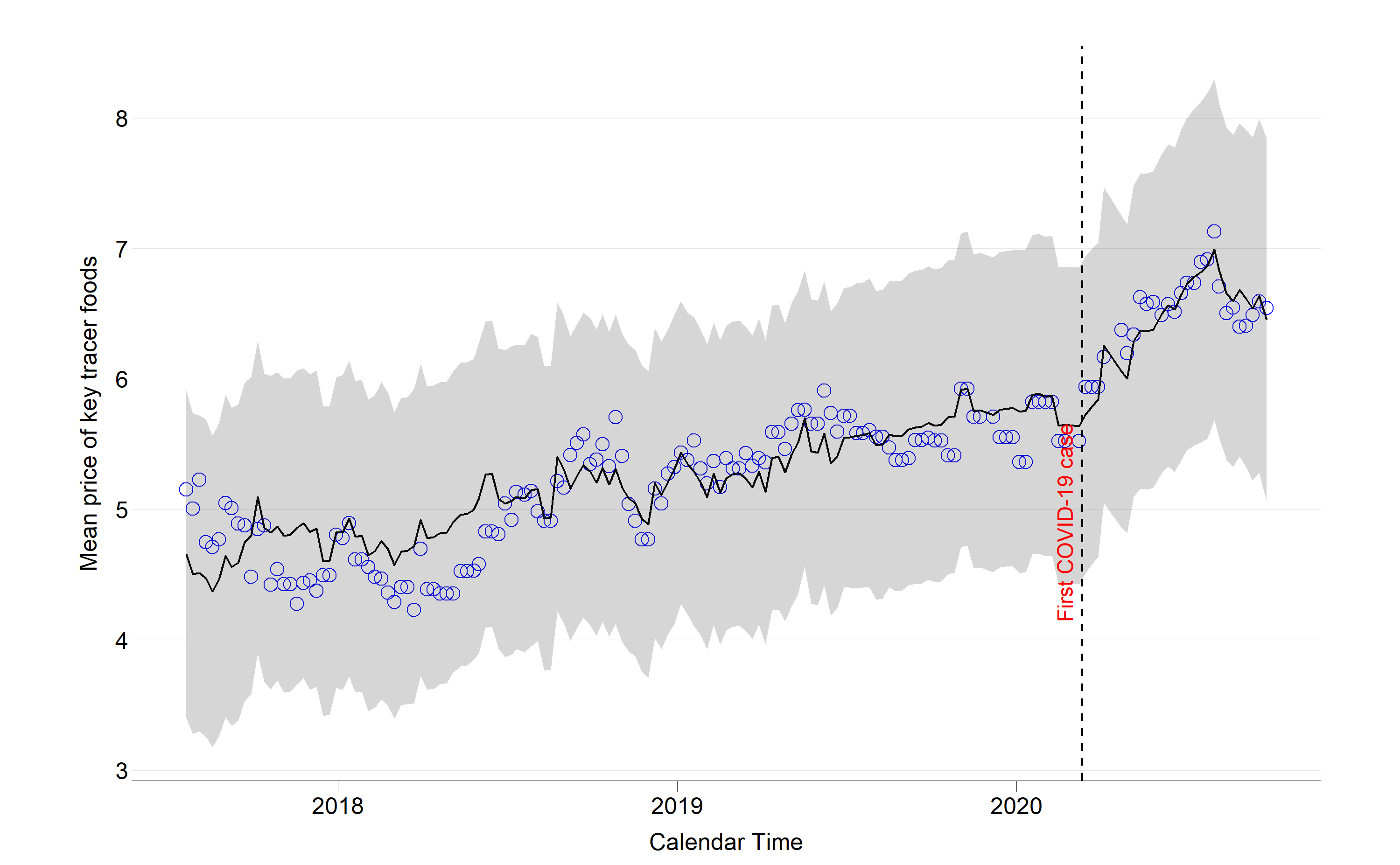


**Supplement file 1. Observed and predicted mean prices of essential food commodities based on the food-group-based model**

Mean predicted price

95% CI

Observation
