# Supplementary material for: Impact of COVID-19 pandemic on food availability and affordability: an interrupted time series analysis in Ghana
Source: BMC Public Health. 2024 May 8;24:1268. doi: 10.1186/s12889-024-18745-x (PMC11080309; doi:10.1186/s12889-024-18745-x)
Supplement: Supplementary file 2 — Supplementary Material 2. [file 12889_2024_18745_MOESM2_ESM.docx]

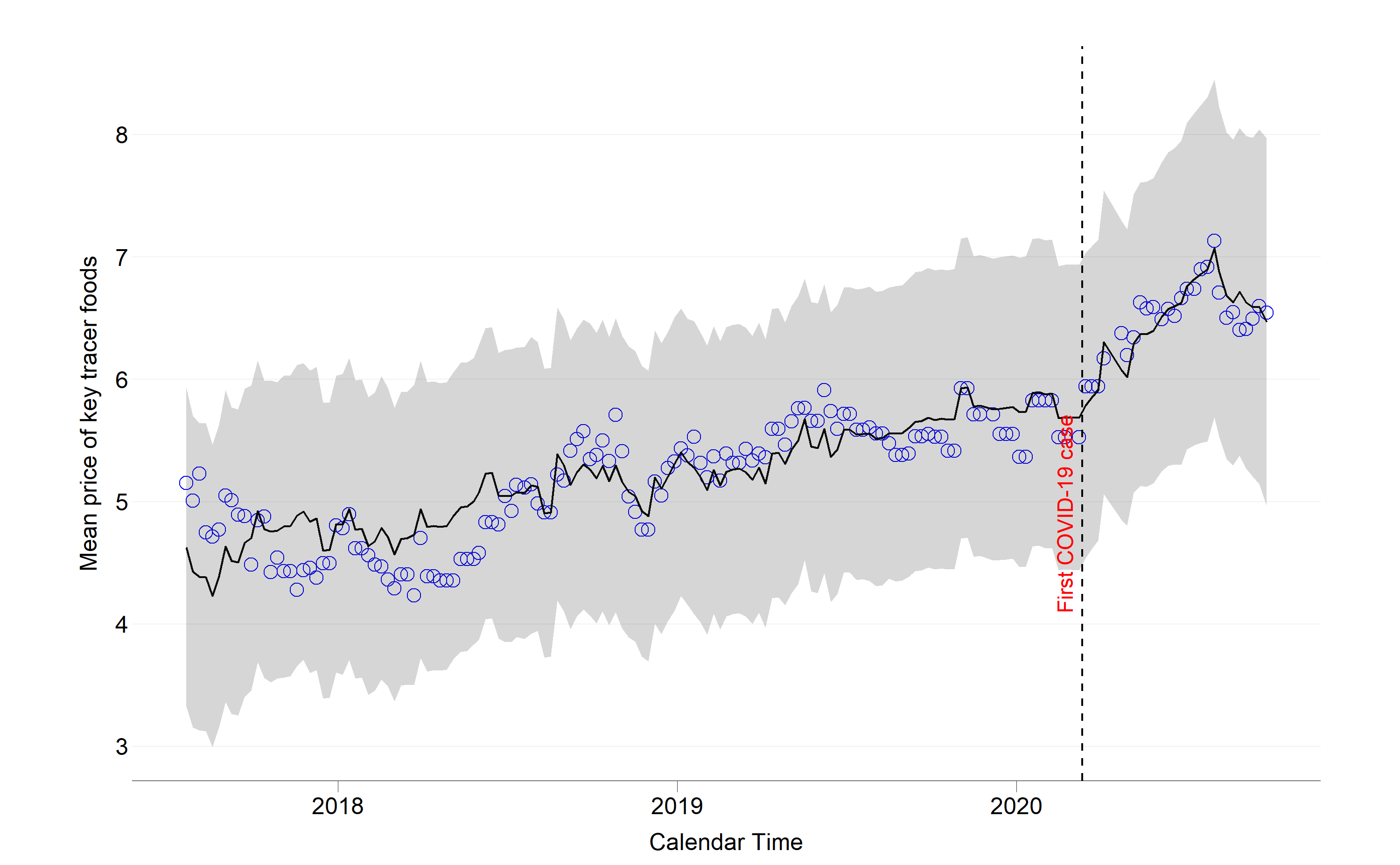


Mean predicted price

**Supplement file 2. Observed and predicted mean prices of essential food commodities based on the region-based model**

95% CI

Observation
