# Supplementary figures and images for: Impact of COVID-19 pandemic on food availability and affordability: an interrupted time series analysis in Ghana
Source: BMC Public Health. 2024 May 8;24:1268. doi: 10.1186/s12889-024-18745-x (PMC11080309; doi:10.1186/s12889-024-18745-x)

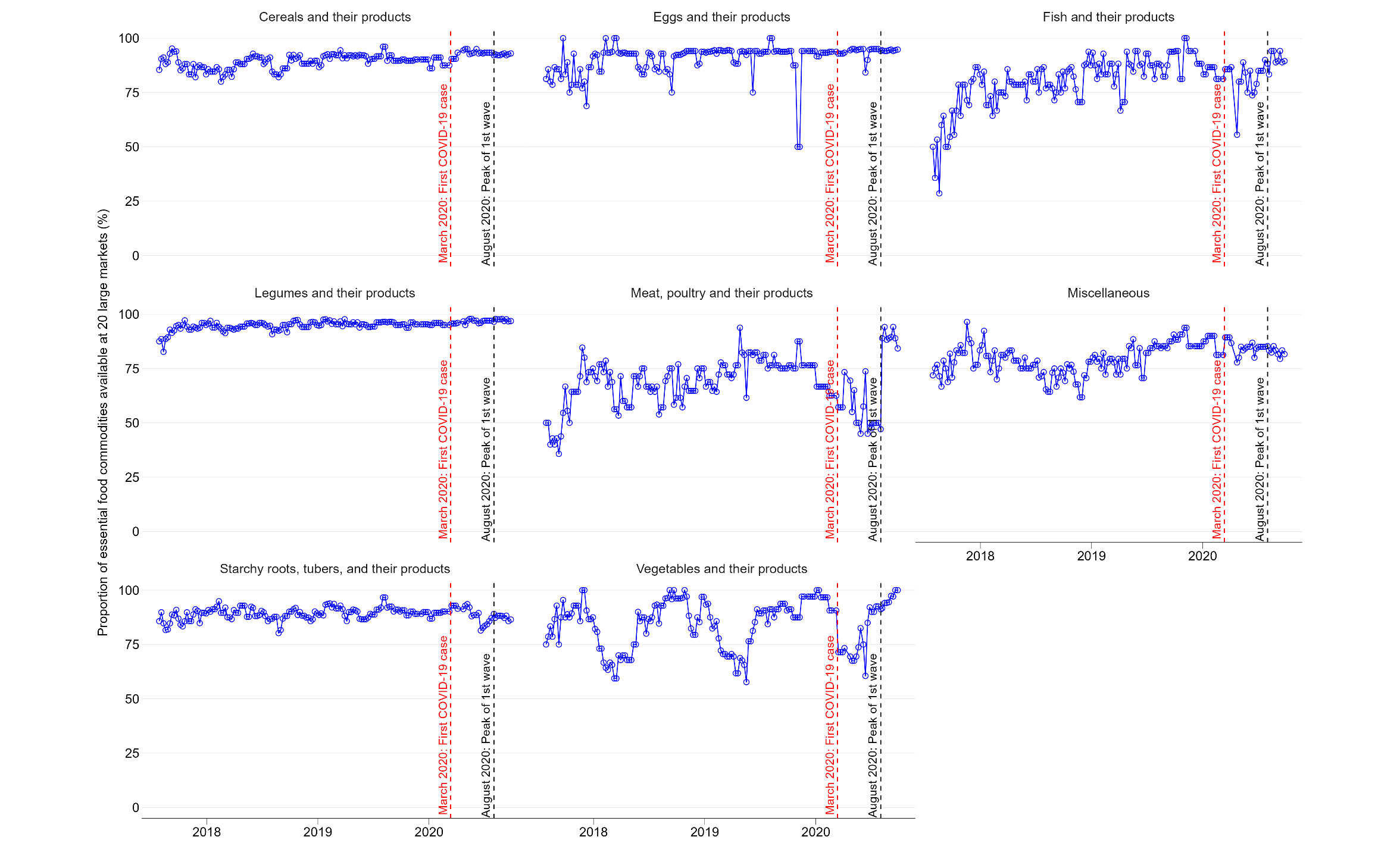


**Supplement file 3: Changes in the proportion of food availability by food group**

Supplement: Supplementary file 3 — Supplementary Material 3. [file 12889_2024_18745_MOESM3_ESM.docx]
