# Supplementary material for: Impact of COVID-19 pandemic on food availability and affordability: an interrupted time series analysis in Ghana
Source: BMC Public Health. 2024 May 8;24:1268. doi: 10.1186/s12889-024-18745-x (PMC11080309; doi:10.1186/s12889-024-18745-x)
Supplement: Supplementary file 4 — Supplementary Material 4. [file 12889_2024_18745_MOESM4_ESM.docx]

| **Supplement file 4.** **Results of interrupted time-series analysis with the interaction between food groups and COVID-19 impacts** | | | | |
| --- | --- | --- | --- | --- |
|  | **Coefficients** | **95% CI** | | **p-value** |
|  |  | **Low** | **High** |  |
| **Time** | 0.002 | -0.002 | 0.005 | 0.340 |
| **Month** |  |  |  |  |
| January | Ref. | - | - | - |
| February | -0.029 | -0.143 | 0.085 | 0.615 |
| March | -0.051 | -0.198 | 0.097 | 0.501 |
| April | -0.012 | -0.175 | 0.152 | 0.887 |
| May | 0.061 | -0.111 | 0.233 | 0.485 |
| June | 0.043 | -0.133 | 0.219 | 0.633 |
| July | 0.039 | -0.138 | 0.217 | 0.664 |
| August | 0.031 | -0.144 | 0.206 | 0.729 |
| September | 0.043 | -0.132 | 0.217 | 0.634 |
| October | 0.035 | -0.136 | 0.206 | 0.688 |
| November | 0.074 | -0.085 | 0.233 | 0.360 |
| December | 0.027 | -0.107 | 0.161 | 0.696 |
| **Region** |  |  |  |  |
| Bono East region | Ref. | - | - | - |
| Ashanti | 0.076 | -1.070 | 1.222 | 0.896 |
| Bono | 0.045 | -1.476 | 1.566 | 0.954 |
| Central | 1.982 | 0.689 | 3.276 | 0.003 |
| Eastern | 0.328 | -1.216 | 1.871 | 0.677 |
| Accra | 1.713 | 0.576 | 2.850 | 0.003 |
| Nothern | -0.005 | -1.267 | 1.258 | 0.994 |
| Upper east | -0.480 | -1.728 | 0.767 | 0.450 |
| Upper west | 0.888 | -0.641 | 2.417 | 0.255 |
| Volta | -0.521 | -1.769 | 0.728 | 0.413 |
| Western | 1.024 | -0.497 | 2.545 | 0.187 |
| Cereal | 1.624 | 0.575 | 2.673 | 0.003 |
| **Food group** |  |  |  |  |
| **Legumes** | Ref. | - | - | - |
| Egg | 0.476 | -1.162 | 2.113 | 0.569 |
| Fish | 7.475 | 5.783 | 9.166 | 0.000 |
| Meat | 7.118 | 5.566 | 8.669 | 0.000 |
| Miscellaneous | 9.285 | 8.016 | 10.553 | 0.000 |
| Starchy | 0.056 | -0.821 | 0.934 | 0.900 |
| Vegetable | 2.381 | 1.128 | 3.634 | 0.000 |
| **Interaction between time and food groups** |  |  |  |  |
| Egg × time | 0.002 | -0.007 | 0.011 | 0.686 |
| Fish × time | 0.028 | 0.019 | 0.038 | 0.000 |
| Cereal × time | 0.003 | -0.003 | 0.009 | 0.346 |
| Meat × time | 0.025 | 0.015 | 0.035 | 0.000 |
| Starchy × time | -0.003 | -0.008 | 0.002 | 0.259 |
| Miscellaneous × time | 0.043 | 0.036 | 0.050 | 0.000 |
| Vegetable × time | -0.001 | -0.008 | 0.006 | 0.729 |
| **Reference group: Legumes and their products** |  |  |  |  |
| COVID1 | 0.004 | -0.030 | 0.039 | 0.813 |
| COVID2 | 0.009 | -0.096 | 0.114 | 0.869 |
| **Interaction between COVID-19 impacts and food groups** |  |  |  |  |
| Cereal × COVID1 | 0.029 | -0.030 | 0.087 | 0.337 |
| Cereal × COVID2 | -0.033 | -0.206 | 0.140 | 0.708 |
| Starchy × COVID1 | 0.123 | 0.078 | 0.168 | 0.000 |
| Starchy × COVID2 | -0.284 | -0.422 | -0.146 | 0.000 |
| Vegetable × COVID1 | 0.041 | -0.023 | 0.106 | 0.208 |
| Vegetable ×COVID2 | -0.075 | -0.270 | 0.120 | 0.453 |
| Meat × COVID1 | -0.068 | -0.153 | 0.016 | 0.114 |
| Meat × COVID2 | 0.037 | -0.218 | 0.292 | 0.775 |
| Egg ×COVID1 | 0.051 | -0.033 | 0.135 | 0.233 |
| Egg×COVID2 | -0.123 | -0.377 | 0.132 | 0.345 |
| Fish × COVID1 | -0.026 | -0.112 | 0.060 | 0.548 |
| Fish × COVID2 | 0.019 | -0.243 | 0.282 | 0.885 |
| Miscellaneous × COVID1 | -0.020 | -0.085 | 0.046 | 0.557 |
| Miscellaneous × COVID2 | -0.067 | -0.269 | 0.136 | 0.517 |
|  | | |  |  |
