# Supplementary material for: Impact of COVID-19 pandemic on food availability and affordability: an interrupted time series analysis in Ghana
Source: BMC Public Health. 2024 May 8;24:1268. doi: 10.1186/s12889-024-18745-x (PMC11080309; doi:10.1186/s12889-024-18745-x)
Supplement: Supplementary file 5 — Supplementary Material 5. [file 12889_2024_18745_MOESM5_ESM.docx]

| **Supplement file 5. Results of interrupted time-series analysis with the interaction between food groups and COVID-19 impacts** | | | | |
| --- | --- | --- | --- | --- |
|  | **Coefficients** | **95% CI** | | **p-value** |
|  |  | **Low** | **High** |  |
| **Time** | 0.007 | 0.002 | 0.013 | 0.006 |
| **Month** |  |  |  |  |
| January | Ref. | - | - | - |
| February | -0.029 | -0.143 | 0.085 | 0.618 |
| March | -0.049 | -0.197 | 0.100 | 0.521 |
| April | -0.008 | -0.172 | 0.157 | 0.927 |
| May | 0.066 | -0.108 | 0.239 | 0.456 |
| June | 0.046 | -0.132 | 0.224 | 0.614 |
| July | 0.038 | -0.141 | 0.218 | 0.675 |
| August | 0.031 | -0.146 | 0.208 | 0.731 |
| September | 0.043 | -0.134 | 0.220 | 0.632 |
| October | 0.037 | -0.135 | 0.209 | 0.675 |
| November | 0.071 | -0.089 | 0.231 | 0.384 |
| December | 0.023 | -0.111 | 0.158 | 0.736 |
| **Region** |  |  |  |  |
| Bono East region | Ref. | - | - | - |
| Ashanti | -0.109 | -1.427 | 1.209 | 0.871 |
| Bono | -0.274 | -1.952 | 1.403 | 0.748 |
| Central | 1.924 | 0.388 | 3.459 | 0.014 |
| Eastern | 1.284 | -0.421 | 2.990 | 0.140 |
| Accra | 1.132 | -0.130 | 2.394 | 0.079 |
| Northern | 0.374 | -1.039 | 1.787 | 0.603 |
| Upper East | 0.028 | -1.354 | 1.410 | 0.968 |
| Upper West | 1.129 | -0.580 | 2.839 | 0.195 |
| Volta | -0.704 | -2.094 | 0.686 | 0.320 |
| Western | 0.926 | -0.758 | 2.609 | 0.281 |
| **Food group** |  |  |  |  |
| Egg | -1.176 | -2.719 | 0.367 | 0.135 |
| Fish | 7.960 | 6.376 | 9.545 | 0.000 |
| Legumes | -1.955 | -2.894 | -1.017 | 0.000 |
| Meat | 7.336 | 6.019 | 8.653 | 0.000 |
| Miscellaneous | 11.170 | 9.963 | 12.378 | 0.000 |
| Starchy | -1.826 | -2.705 | -0.947 | 0.000 |
| Vegetable | 0.438 | -0.762 | 1.639 | 0.474 |
| **Interaction between time and regions** |  |  |  |  |
| Time × Ashanti | 0.001 | -0.007 | 0.008 | 0.860 |
| Time × Bono | 0.005 | -0.004 | 0.014 | 0.285 |
| Time × Central | 0.000 | -0.010 | 0.009 | 0.918 |
| Time × Eastern | -0.014 | -0.023 | -0.005 | 0.003 |
| Time × Accra | 0.005 | -0.002 | 0.012 | 0.162 |
| Time × Northern | -0.004 | -0.013 | 0.005 | 0.361 |
| Time × Upper East | -0.007 | -0.015 | 0.001 | 0.072 |
| Time × Upper West | -0.002 | -0.011 | 0.008 | 0.747 |
| Time × Volta | 0.004 | -0.003 | 0.012 | 0.255 |
| Time × Western | 0.000 | -0.009 | 0.009 | 0.990 |
| **Reference group: Bono East region** |  |  |  |  |
| COVID1 | 0.026 | -0.025 | 0.077 | 0.320 |
| COVID2 | -0.058 | -0.214 | 0.099 | 0.471 |
| **Interaction between COVID-19 impacts and Regions** |  |  |  |  |
| Ashanti × COVID1 | 0.041 | -0.026 | 0.108 | 0.230 |
| Ashanti × COVID2 | -0.065 | -0.278 | 0.147 | 0.546 |
| Bono × COVID1 | -0.029 | -0.116 | 0.058 | 0.509 |
| Bono × COVID2 | -0.002 | -0.269 | 0.264 | 0.987 |
| Central × COVID1 | 0.052 | -0.028 | 0.131 | 0.204 |
| Central × COVID2 | -0.193 | -0.419 | 0.033 | 0.094 |
| Eastern × COVID1 | 0.108 | 0.019 | 0.197 | 0.017 |
| Eastern × COVID2 | -0.142 | -0.416 | 0.133 | 0.313 |
| Greater Accra × COVID1 | 0.075 | 0.009 | 0.141 | 0.026 |
| Greater Accra × COVID2 | -0.235 | -0.436 | -0.033 | 0.023 |
| Northern × COVID1 | 0.017 | -0.065 | 0.098 | 0.689 |
| Northern × COVID2 | 0.006 | -0.230 | 0.241 | 0.963 |
| Upper East × COVID1 | -0.002 | -0.078 | 0.073 | 0.953 |
| Upper East × COVID2 | 0.231 | 0.007 | 0.455 | 0.043 |
| Upper West × COVID1 | -0.004 | -0.095 | 0.086 | 0.925 |
| Upper West × COVID2 | -0.083 | -0.365 | 0.198 | 0.563 |
| Volta × COVID1 | -0.063 | -0.136 | 0.011 | 0.095 |
| Volta × COVID2 | 0.094 | -0.129 | 0.317 | 0.410 |
| Western × COVID1 | 0.030 | -0.057 | 0.117 | 0.499 |
| Western × COVID2 | -0.017 | -0.285 | 0.252 | 0.903 |
|  | | |  |  |
