# Supplementary material for: Impact of COVID-19 pandemic on food availability and affordability: an interrupted time series analysis in Ghana
Source: BMC Public Health. 2024 May 8;24:1268. doi: 10.1186/s12889-024-18745-x (PMC11080309; doi:10.1186/s12889-024-18745-x)
Supplement: Supplementary file 6 — Supplementary Material 6. [file 12889_2024_18745_MOESM6_ESM.docx]

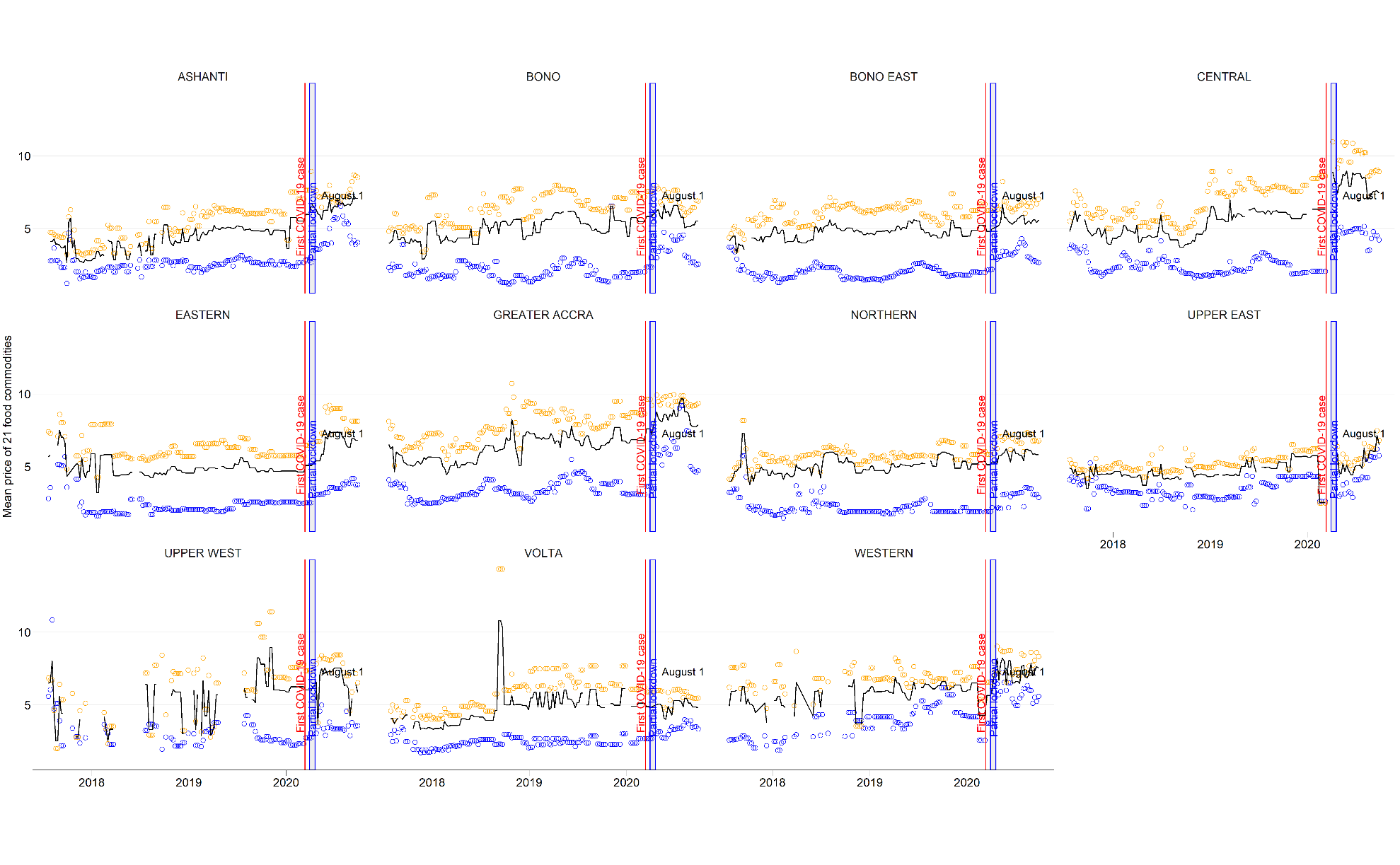


**Supplement file 6. Changes in mean observed prices of the starchy food group and other food groups by region**

Mean prices of all tracer food commodities

Mean food prices other than Starchy good

Mean prices of Starchy food group
